# Diagnostic Utility and Psychometric Properties of the Beck Depression Inventory-II Among Korean Adults

**DOI:** 10.3389/fpsyg.2019.02934

**Published:** 2020-01-21

**Authors:** Kiho Park, Eunju Jaekal, Seowon Yoon, Seung-Hwan Lee, Kee-Hong Choi

**Affiliations:** ^1^Department of Psychology, Korea University, Seoul, South Korea; ^2^KU Mind Health Institute, Korea University, Seoul, South Korea; ^3^Department of Psychiatry, Inje University Ilsanpaik Hospital, Goyang, South Korea; ^4^Clinical Emotion and Cognition Research Laboratory, Inje University, Goyang, South Korea

**Keywords:** BDI-II, cutoff, validation, diagnostic utility, depressive disorders, screening tool

## Abstract

The Beck Depression Inventory-II (BDI-II) is one of the most widely used depression assessment tools in Korea. However, the psychometric properties and diagnostic cut-off point of the official Korean version of the BDI-II have not yet been reported. This study aims to clarify the psychometric properties and diagnostic utility of the Korean BDI-II. A total of 1,145 clinical and non-clinical Korean adults participated in this study. The BDI-II showed a high level of internal consistency and high correlations with other depression-related measures. Confirmatory factor analysis (CFA) was performed, and a 3-factor model showed the best model fit. To identify the diagnostic utility of the BDI-II, the Quality Assessment of Diagnostic Accuracy Studies 2nd Edition (QUADAS-2) methodology was applied in participant recruitment and research design. Results of ROC curve analysis suggested two optimal cut-off scores, 23 points for detecting major depressive disorder (MDD) (83.3% sensitivity, 86.8% specificity) and 17 points for depressive-related disorder (80.9% sensitivity, 76.4% specificity). To identify the usefulness of the BDI-II as a severity assessment tool or screening tool, a test information curve (TIC) was generated with an Item Response Theory (IRT) analysis. The TIC was flat and plateau-like, indicating its appropriateness as a severity rating tool. Research data supports the BDI-II as a reliable and valid screening tool as well as a severity rating tool in the Korean adult population.

## Introduction

Major depressive disorder (MDD) is one of the most common psychiatric disorders, characterized by depressed mood or loss of interest in daily life (American Psychiatric Association [APA], 2013). According to the World Health Organization (WHO), the global prevalence of MDD in 2015 was estimated at 4.4% (322 million people) (World Health Organization [WHO], 2017). The Ministry of Health and Welfare of South Korea reported a similar figure (approximately 5.0%) (Ministry of Health and Welfare, 2016). Recently, greater attention has been paid to the MDD epidemic in Korea due to the high suicide rate, the second highest among the Organization for Economic Cooperation and Development (OECD) countries. In 2015, Korea had a suicide rate of 25.8 per 100,000 people, far above the OECD average of 11.6 (OECD, 2019) According to one systematic review of psychological autopsy studies of suicide, 91% of people who committed suicide suffered from psychiatric problems, of which the most common were depressive disorders (Cavanagh et al., 2003).

The US Preventive Services Task Force (USPTF) recommended early detection and screening for depressive disorders in primary care settings (Siu et al., 2016). A meta-analysis study also reported that the shorter the duration of untreated illness (DUI), the better the prognosis in the following treatment course (Ghio et al., 2014). Despite the importance of early screening and treatment of depressive disorders, less than half (approximately 40.4%) the people diagnosed with mood disorders received psychiatric services (Ministry of Health and Welfare, 2016). Under these circumstances, having a proper screening tool for depressive disorders is a prerequisite to enhancing awareness of the severity of depression and accessibility to optimal treatment.

The Beck Depression Inventory-II (BDI-II) (Beck et al., 1996) is one of the most widely used screening tools for depressive disorders and is also used to measure the severity of depression (McDowell, 2006). The BDI-II has been translated into various languages and applied in numerous countries. According to a comprehensive review of the psychometric properties of the BDI-II using 118 studies conducted with 60,126 participants worldwide from 1996 to 2013, the BDI-II can be regarded as a cost-effective tool to measure the severity of depression, which is widely applicable for both research and clinical settings worldwide (Wang and Gorenstein, 2013).

Although the BDI-II was originally developed to reflect and monitor the severity of depression over the course of illness and treatment (Beck et al., 1961), it has been demonstrated to be a useful screening tool with optimal cut-off scores. However, the cut-off scores recommended by multiple studies screening depressive disorders showed large variances for different populations. For instance, seven points for MDD screening for Parkinson’s disease (Williams et al., 2012), 10 points for depressive disorders (MDD, dysthymic disorder, and depressive disorder not otherwise specified) among undergraduate students who are taking an introductory psychology class (Shean and Baldwin, 2008), and 24 points for MDD among psychiatric inpatient adolescents (Krefetz et al., 2002). Methodological differences between studies result in varying recommendations of cut-off scores. For instance, Osman et al. (2008) separately recruited high school students and inpatients samples, and calculated cut-off scores distinguishing the two groups. Given the extreme differences in characteristics between the groups, diagnostic functions and cut-off scores of BDI-II should differ from those employing random sampling methods.

From this perspective, to assure the quality of diagnostic accuracy studies, Whiting et al. (2011) suggested the Quality Assessment of Diagnostic Accuracy Studies 2nd Edition (QUADAS-2), which presents specific norms for subject recruitment and selection, index test, conditions for reference standard, research procedure, and timing. For subject recruitment and selection, first, the QUADAS-2 evaluates whether participants are enrolled randomly and consecutively, and whether the study inappropriately excludes samples like “difficult-to-diagnose” patients. Second, it evaluates whether the index test (e.g., BDI-II) results are interpreted without knowledge of the results of the reference standard (e.g., diagnostic interview), or vice versa. Third, a selected reference standard should be considered when evaluating the quality of diagnostic accuracy. For example, when evaluating the diagnostic power of BDI-II, if the reference standard is a CES-D or DSM-5 diagnosis confirmed with a structured interview, the latter should provide more accurate information. Finally, for the procedure and assessment orders, the index test and the reference standard should be conducted in similar time frames. If the reference standard test was done several months before the index test, some study participants might have remitted from the mental health condition.

Among 24 studies reporting an optimal cut-off score of BDI (Wang and Gorenstein, 2013), only two studies conducted in the United States were in accordance with the criteria that the QUADAS-2 suggests: One study conducted with 340 primary care medical patients reported 18 as a cut-off score (Arnau et al., 2001), while another study with 220 African American primary care patients reported 14 as a cut-off score for BDI-II (Dutton et al., 2004). Considering that the psychometric properties of questionnaires are closely related to the race and culture of the population to which it will be applied (Iwata and Buka, 2002), it is crucial to verify the diagnostic and psychometric properties of the BDI-II in samples of the countries where it is used.

Several studies have validated the BDI-II in Korean samples, and two reported cut-off points for screening MDD (Lim et al., 2011) or depressive disorders (Sung et al., 2008). However, neither study fully satisfied the QUADAS-2 criteria. First, both studies recruited clinical and control groups separately. When recruiting the control group, participants with no psychiatric history (e.g., college students or hospital staff) were pre-selected even before conducting a diagnostic interview, i.e., reference standard. This research procedure resulted in excluding “difficult-to-diagnose” participants who would experience mild levels of depressive symptoms with remission or those without past depressive disorders, and thus artificially increase the discriminability of the screening tool between the clinical group and control group. Second, since the positive rate of the data set was too high (due to a smaller control sample size), overestimated predictions by the screening tool may have led to good discrimination power in the ROC curve analysis (Lobo et al., 2008). Third, in Sung et al.’s (2008) study, the Hamilton depression rating scale (HDRS) was used as a diagnostic criterion. However, the HDRS is not recommended for use as a diagnostic index test (Hamilton, 1967). Finally, even though the Korean version BDI-II was officially translated and published in 2014 with a full license (Lee et al., 2017), it has only been validated for adolescent populations without optimal cut-off scores. Therefore, it is timely and necessary to validate the Korean version BDI-II for the Korean adult population and examine its diagnostic properties as a screening tool.

In addition, although BDI-II was originally developed and validated as a depression severity measure (Cameron et al., 2011; Titov et al., 2011), it has also been used as a screening tool (Zich et al., 1990). In the current study, we investigated the usefulness of BDI-II as a severity assessment tool or screening tool using Item Response Theory (IRT).

Therefore, the purposes of this study were (1) to examine the psychometric properties (e.g., reliability, factor structures, other construct validity) and diagnostic screening utility with optimal cutoff scores of the BDI-II as a screening tool within the framework of QUADAS-2, and (2) to investigate using IRT whether BDI-II would be more appropriate as a severity or screening tool.

## Materials and Methods

### Participants

The current study was a part of an umbrella project, entitled “The Development of Korean Depression and Anxiety Screening Scale.” A total of 1,145 adult participants were recruited from two different settings. First, 555 participants were randomly recruited through online advertisements. The remaining 590 participants were recruited among visitors at hospitals using the consecutive sampling method from two different general hospitals. Thus, participants recruited from the hospitals included clinical (e.g., psychiatric and non-psychiatric patients) and non-clinical samples (e.g., patients’ families, friends, visitors, and hospital staff members). Researchers were blind to medical charts of participants with psychiatric conditions, and thus, conducted individual diagnostic interviews and psychological tests without knowing their medical diagnosis. Individual psychiatric diagnostic interviews and psychological tests were conducted at research labs in the university or the hospitals. Consistent with our aims that investigated clinical utility of the BDI-II in real-world community mental health settings and medical or primary care settings, minimum inclusion criteria and exclusion criteria were established. All adults over 18 years were included in the inclusion criteria. Participants who were not fluent in Korean or illiterate were excluded from the current study. All participants in this research voluntarily participated after providing written informed consent forms. This study was approved by the local institutional review boards. Detailed demographic information is presented in Table 1.

**TABLE 1:** Sample demographics.

	Online advertisement sample (*N* = 555)	Hospital visitor sample (*N* = 590)	
	*M (SD)*	*M (SD)*	*t*
Age	31.7 (12.3)	41.6 (15.0)	−12.10^∗∗∗^
Education(years)	14.6 (2.4)	14.6 (3.4)	0.03
Depression symptom (BDI-II score)	13.07 (9.8)	14.22 (13.0)	−1.68

	****N* (%)***	****N* (%)***	**χ*2***

**Gender**			
Female	354 (63.8)	409 (69.3)	4.65^∗^
Unreported	–	4 (0.7)	
**Marital status**			
Single	411 (74.1)	237 (40.2)	123.87^∗∗∗^
Married	135 (24.3)	302 (51.2)	
Divorced	3 (0.5)	18 (3.1)	
Widowed	6 (1.1)	17 (2.9)	
Unreported	–	16 (2.7)	

*^∗^<0.05, ^∗∗∗^<0.001.*

### Procedure

To evaluate its usefulness as a diagnostic tool, the methodology presented in QUADAS-2 (Whiting et al., 2011) was applied in this study. The QUADAS-2 framework comprises four domains. The first domain is patient selection, which is intended to prevent only biased samples from being included in the study. To avoid selection bias (e.g., deliberately excluding difficult-to-diagnose patients), this study included all difficult-to-diagnose patients and recruited participants regardless of their diagnosis, rather than comparing selective samples from psychiatric patients and healthy university students. The second domain is whether the evaluator is affected by the results of the reference test in conducting the index test. To prevent researcher’s bias, testing was conducted blind to other reference test results and psychiatric diagnosis. That is, interviewers who conducted the diagnostic interviews were not aware of either psychological test results such as BDI-II and CES-D, or their medical records. The third domain is the adequacy of the reference standard. Psychiatric diagnosis obtained from a structured diagnostic interview tool, the Mini-International Neuropsychiatric Interview-Plus (M.I.N.I.), was utilized as a reference standard. The diagnostic interview was conducted by psychiatrists, licensed clinical psychologists, and clinical psychology graduate students supervised by licensed psychologists and a psychiatrist. The fourth domain is concerned with whether there is a time difference between the index test and the reference standard. The BDI-II and M.I.N.I. diagnostic interviews were performed at the same time.

### Measures

#### Beck Depression Inventory-II Korean Version

The BDI-II is a measure of depression developed by Beck et al. (1996) comprising 21 items measuring depressive symptoms among the emotional, cognitive, motivational, and physiological domains of depression. Each item is scored on a 4-point Likert scale ranging from 0 to 3, total score ranges from 0 to 63. Consistent with the original BDI-II, in the Korean version of the BDI II, respondents select one of four statements that best describe how they felt during the last 2 weeks. Higher scores indicate that respondents’ depressive symptoms are more severe. In this study, K-BDI-II, which has been published in Korean, was used (Lee et al., 2017). In this study by Lee et al. (2017), two independent licensed clinical psychologists translated the original English version of the BDI-II into Korean with the permission of the publisher, The Psychological Corporation. After, three researchers confirmed the questionnaire content through a debate, it was re-translated into English by a proficient bilingual person with a master’s degree in clinical psychology. Researchers reviewed and revised the final version of the K-BDI-II.

#### The Mini-International Neuropsychiatric Interview-Plus (M.I.N.I.)

The Mini-International Neuropsychiatric Interview-Plus is a structured interview tool developed for the diagnosis of major axis 1 mental disorders from the ICD-10 (International Classification of Diseases-10th Revision) and DSM-IV (Sheehan et al., 1998). In this study, a translated version of the M.I.N.I was used, and diagnostic accuracy was reported for the Korean version of the M.I.N.I. (Yoo et al., 2006). Specifically, Kappa statistics for MDD and bipolar disorder were 0.71 and 0.74, respectively (Yoo et al., 2006).

#### Center for Epidemiologic Studies Depression Scale Korean Version

Center for Epidemiologic Studies Depression Scale (CES-D) was developed by Radloff to measure depressed levels in 1977 (Radloff, 1977). CES-D is a 20-item self-report scale that measures the frequency of depression experienced during the past week on four levels. The total score is 60 points and the higher the score, the greater the severity of depression. This study used the Korean version of CES-D, which was verified and validated in Korean (Cho and Kim, 1993). The test-retest reliability was 0.68 for non-clinical samples and 0.83 for clinical samples. Additionally, a score of 25 was presented as an optimal cut-off score, with sensitivity = 0.93 and specificity = 0.79 (Cho and Kim, 1993).

#### Patient Health Questionnaire-9 Korean Version

Patient health questionnaire-9 (PHQ-9) is a depression scale developed by Kroenke et al. (2001). PHQ-9 measures nine areas including unpleasantness, depression, sleep changes, fatigue, appetite change, guilt, unreasonableness, loss of concentration, depressed feeling, and suicidal thoughts that occurred during the past 2 weeks. It is scored from 0 (*not at all*) to three points (*almost everyday*), and the maximum total score is 27 points. The higher the score, the greater the severity of depression. In 2010, a study of the validity and reliability of the PHQ-9 Korean version was conducted (Park et al., 2010). In this study, the PHQ-9 Korean version was used.

#### Generalized Anxiety Disorder 7-Item (GAD-7) Korean Version

The GAD-7 is a simple self-report assessment tool designed to screen for generalized anxiety disorder (GAD) and to measure the severity of its symptoms. Subjects are asked to report the frequency of anxiety symptoms over the past 2 weeks using a 4-point Likert scale. The Korean version of the GAD-7 (Pfizer, 2018; Ahn et al., 2019), which is presented on the Patient Health Questionnaire website^1^, was utilized in this study.

### Statistical Analysis

The IBM SPSS Statistics 23 program was used to perform descriptive statistics, correlational analysis, and ROC curve analysis. To perform confirmatory factor analysis (CFA), MPLUS software 7.0 (Muthén and Muthén, 2012) was used. To evaluate model fit, incremental fit indices, such as the Tucker-Lewis Index (TLI) and Comparative Fit Index (CFI), absolute model fit indices such as the model chi-square (χ^2^), Root Mean Square Error of Approximation (RMSEA), and Standardized Root Mean Squared Residual (SRMR), and information criteria such as Akaike’s Information Criterion (AIC), Bayesian Information Criteria (BIC), and sample-size-adjusted BIC (aBIC) were used. These model fit indices were interpreted following standard criteria, including CFI and TLI exceeding 0.95 and RMSEA lower than 0.08 (Bentler, 1990). Values of SRMR of 0.08 or lower (Hu and Bentler, 1999) also indicated good model fit. Finally, the lower the information criteria values, the better the model fit (Akaike, 1987).

IRT analysis was performed using the “mirt” package (Chalmers, 2012) for the R statistical program (version 3.5.0). The graded response model (GRM) was applied for analysis. GRM is one of the IRT models appropriate for ordered polytomous categories like Likert scales (Samejima, 1970). IRT analysis provides the Test Information Curve (TIC), which depicts the amount of information yielded by the test at given ability level. If the TIC is evenly distributed on the *x*-axis of θ, which refers to the level of the domain being measured, it is an appropriate test to measure all ranges of ability levels like the Scholastic Aptitude Test. Such a shape would be more appropriate for measuring the severity of depression. On the other hand, if the test is designed to award scholarships, more accuracy is required for ability levels near the cut-off. The best TIC in this situation would peak at the cut-off score point (Baker and Kim, 2004). Therefore, it was possible to assess the suitability of the test for a certain purpose according to the shape of the TIC.

## Results

### Prevalence of Depressive Symptoms

The average BDI-II total score for all participants was 13.66 (*SD* = 11.54). In total, 472 (41.2% of the sample) participants scored 14 or over, indicating mild levels of depression. The mean and standard deviation for each item and total score are presented in Table 2. By using M.I.M.I psychiatry structured interviews, 96 (8.4%) were diagnosed with MDD and 188 (16.4%) were classified with depressive-related disorder (DD), which includes MDD, dysthymia, past MDD currently in partial remission, past MDD current in full remission but still on medication, and depressive disorder not otherwise specified. Since DD is a broader concept than MDD, DD includes the number of patients diagnosed with MDD. Among all participants with depressive-related disorder, 126 were comorbid with other psychiatric disorders like anxiety disorder. Among all participants, 676 (59%) were not diagnosed with any past or current disorder and were classified as the “healthy” group.

**TABLE 2:** Mean, standard deviations, and item-total correlations of the Korean *BDI-II*.

Item	DD (*N* = 188)	Control (*N* = 957)	Total (*N* = 1,145)	*r*_tot_	Cronbach’s α if item is deleted
	*M (SD)*	*M (SD)*	*M (SD)*		
1. Sadness	1.15 (0.859)	0.41 (0.560)	0.53 (0.676)	0.753^∗∗∗^	0.942
2. Pessimism	1.42 (0.993)	0.57 (0.694)	0.71 (0.813)	0.735^∗∗∗^	0.942
3. Past failure	1.38 (0.960)	0.46 (0.689)	0.61 (0.813)	0.732^∗∗∗^	0.943
4. Loss of pleasure	1.49 (0.956)	0.67 (0.717)	0.8 (0.819)	0.759^∗∗∗^	0.942
5. Guilty Feeling	1.34 (1.034)	0.65 (0.717)	0.76 (0.818)	0.659^∗∗∗^	0.944
6. Punishment feelings	1.38 (1.233)	0.38 (0.758)	0.54 (0.93)	0.724^∗∗∗^	0.943
7. Self-dislike	1.32 (1.012)	0.40 (0.723)	0.55 (0.847)	0.754^∗∗∗^	0.942
8. Self-criticalness	1.39 (1.046)	0.50 (0.792)	0.64 (0.9)	0.743^∗∗∗^	0.942
9. Suicidal thoughts	0.93 (0.777)	0.24 (0.458)	0.36 (0.581)	0.655^∗∗∗^	0.944
10. Crying	1.13 (1.038)	0.39 (0.689)	0.51 (0.805)	0.661^∗∗∗^	0.944
11. Agitation	1.06 (0.948)	0.32 (0.543)	0.44 (0.684)	0.681^∗∗∗^	0.943
12. Loss of interest	1.56 (1.005)	0.61 (0.691)	0.76 (0.83)	0.758^∗∗∗^	0.942
13. Indecisiveness	1.24 (0.899)	0.57 (0.657)	0.68 (0.745)	0.673^∗∗∗^	0.943
14. Worthlessness	1.25 (1.002)	0.32 (0.599)	0.47 (0.761)	0.762^∗∗∗^	0.942
15. Loss of Energy	1.53 (0.825)	0.76 (0.668)	0.88 (0.752)	0.728^∗∗∗^	0.943
16. Changes in sleeping	1.66 (0.985)	0.85 (0.795)	0.98 (0.88)	0.611^∗∗∗^	0.945
17. Irritability	1.19 (0.971)	0.47 (0.647)	0.59 (0.758)	0.690^∗∗∗^	0.943
18. Changes in appetite	1.33 (0.933)	0.62 (0.712)	0.74 (0.795)	0.601^∗∗∗^	0.945
19. Concentration difficulty	1.27 (0.838)	0.57 (0.630)	0.68 (0.716)	0.723^∗∗∗^	0.943
20. Tiredness	1.41 (0.906)	0.66 (0.616)	0.79 (0.727)	0.711^∗∗∗^	0.943
21. Loss of interest in sex	1.35 (1.161)	0.49 (0.777)	0.63 (0.908)	0.546^∗∗∗^	0.946
BDI-II total	27.65 (13.543)	10.92 (8.800)	13.66 (11.54)	–	–

*^∗∗∗^<0.001, DD, Depressive related disorder.*

### Internal Consistency and Convergent Validity

Cronbach’s alpha coefficient for internal consistency was 0.946, indicating a high level of internal reliability. Furthermore, the coefficients of Cronbach’s alpha ranged from 0.942 to 0.946 if individual items were deleted, suggesting that there is no significant benefit from excluding any individual items (Table 2). Means, standard deviations, and item-total correlations are presented in Table 2. Item-total correlations ranged from 0.546 to 0.762, which also indicates good internal consistency. To examine convergent validity, a correlational analysis was conducted, and its coefficients are presented in Table 4. The BDI-II total score was significantly correlated with the PHQ-9 total score (*r* = 0.853, *p* < 0.001) and CES-D total score (*r* = 0.862, *p* < 0.001), indicating good convergent validity. BDI-II also showed a significant correlation with the GAD-7 total score (*r* = 0.797, *p* < 0.001), a screening tool for generalized anxiety disorder (GAD) known to be closely related to depression.

### Factor Structure

**FIGURE 1:**
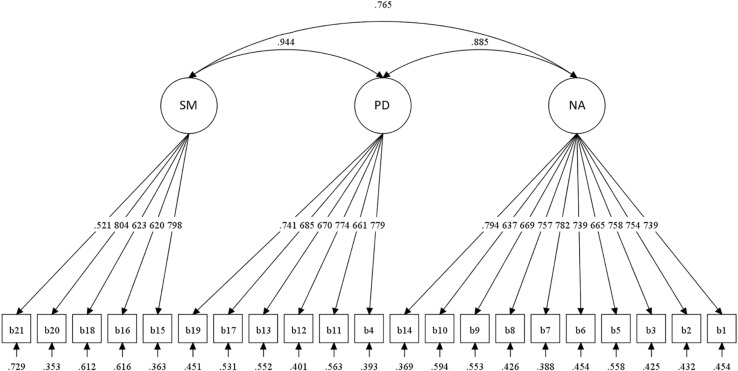
Diagram of confirmatory factor analysis for 3-factor model. SM, Somatic Factor; PD, Performance Difficulty Factor; NA, Negative Attitude Factor.

CFA was performed to examine the factor structure of BDI-II. Traditionally, Beck suggested a 2-factor model (a somatic-affective factor for items 4, 10–13, and 15–21, and a cognitive factor for items 1–3, 5–9, and 14) (Beck et al., 1996). However, Osman et al. (1997) proposed a 3-factor model (negative attitude factor for items 1–3, 5–10, and 14, performance difficulty factor for items 4, 11–13, 17, and 19, and somatic factor for items 15, 16, 18, 20, and 21) that showed greater fit than the 2-factor model. This result was replicated among Asian populations such as Taiwanese adolescents (Wu and Huang, 2014) and Korean adolescent samples (Lee et al., 2017). In this study, Beck’s 2-factor model and 3-factor model were both tested, and the 3-factor model showed a better model fit than the 2-factor model. Summary of Goodness-of-fit Indices for CFA is presented in Table 3 (Results from separate analyses between online advertisement sample and hospital visitor sample are presented in Supplementary Tables S1, S2). The 3-factor model and its factor loadings are depicted in Figure 1. Correlational coefficients between BDI-II total score and the three sub-factors are presented in Table 4.

**TABLE 3:** Summary of Goodness-of-fit Indices for CFA.

	Fit indices								
Model tested	χ*^2^*	AIC	BIC	aBIC	CFI	TLI	SRMR	RMSEA	90% CI
3-Factor Model	814.448^∗∗∗^ (*df* = 186)	43860.58	44193.49	43983.85	0.953	0.947	0.033	0.054	0.051–0.058
2-Factor Model	979.099^∗∗∗^ (*df* = 188)	44021.23	44344.05	44140.77	0.941	0.934	0.038	0.061	0.057–0.064

*AIC, Akaike information criterion; BIC, Bayesian information criterion; aBIC, Sample-size adjusted BIC; CFI, Comparative fit index; TLI, Tucker-Lewis Index; SRMR, Standardized root mean squared residual; RMSEA, Root mean square error of approximation; CI, Confidence interval. ^∗∗∗^<0.001.*

**TABLE 4:** Correlation coefficients of the BDI-II total score with BDI-II sub-factors.

	SM	PD	NA	BDI-II Total
SM	–			
PD	0.779^∗∗∗^	–		
NA	0.671^∗∗∗^	0.794^∗∗∗^	–	
BDI-II total	0.851^∗∗∗^	0.926^∗∗∗^	0.943^∗∗∗^	–

*^∗∗∗^<0.001. SM, Somatic Factor; PD, Performance Difficulty Factor; NA, Negative Attitude Factor.*

### Criterion Validity

**FIGURE 2:**
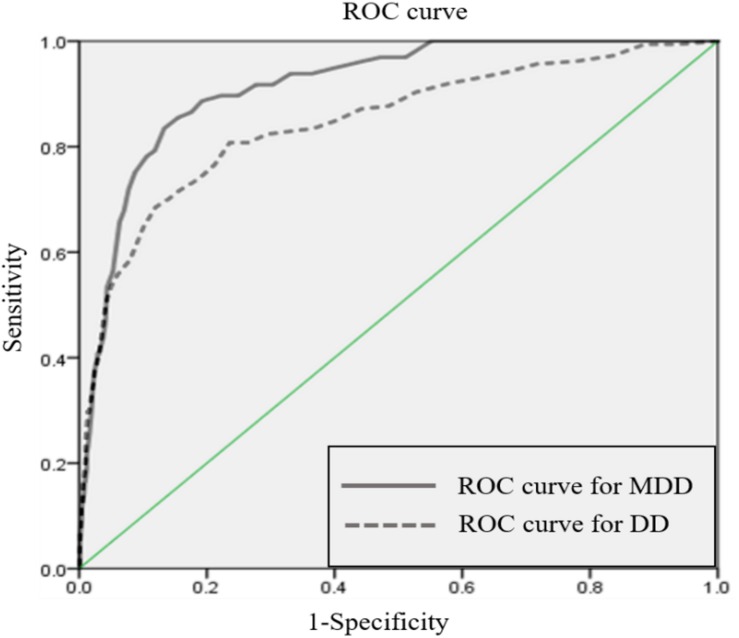
ROC curve for MDD and DD.

To test the criterion validity of BDI-II, ROC analyses were conducted to detect either MDD or depressive disorder. The ROC curves are shown in Figure 2. Area under curve (AUC) for detecting MDD was 0.915 and for detecting depressive related disorder it was 0.846. To calculate optimal cut-off points, Youden’s index (Youden’s index *J* = sensitivity + specificity – 1) (Youden, 1950) was applied. A score of ≥23 was identified as the optimal cut-off score to detect MDD. At this cut-off score, BDI-II screened MDD patients with 0.833 sensitivity, 0.868 specificity, 0.365 positive predictive value (PPV), and 0.983 negative predictive value (NPV). To detect depressive disorder patients, a score of ≥17 was identified as an optimal cut-off score with sensitivity 0.809, specificity 0.764, PPV 0.402, and NPV 0.953. Sensitivity and specificity were calculated with the traditional cut-off scores from Beck et al. (1996) (mild = 14, moderate = 20, severe = 29). Detailed results of the ROC analyses are presented in Table 5.

**TABLE 5:** Results of ROC analyses for the MDD and DD.

	Diagnosis for MDD					Diagnosis for DD				
*n* cases/*n* controls	96/1049					188/957				
AUC (95% CI)	0.915 (0.889–0.941)					0.846 (0.813–0.880)				
Cut off	SEN	SPE	*J*	PPV^b^	NPV	SEN	SPE	*J*	PPV	NPV
MDD optimal cut off ^a^ = 23	**0.833**	**0.868**	**0.701**	**0.365**	**0.983**	0.649	0.899	0.548	0.557	0.929
DD optimal cut off = 17	0.917	0.724	0.641	0.233	0.99	**0.809**	**0.764**	**0.573**	**0.402**	**0.953**
BDI-II mild = 14	0.938	0.636	0.574	0.191	0.991	0.830	0.670	0.5	0.331	0.953
BDI-II moderate = 20	0.885	0.808	0.693	0.297	0.987	0.718	0.842	0.56	0.472	0.938
BDI-II severe = 29	0.656	0.937	0.593	0.488	0.968	0.484	0.960	0.444	0.705	0.905

*AUC, area under curve; CI, confidence interval; MDD, major depressive disorder; DD, depressive related disorder; SEN, sensitivity; SPE, specificity; *J*, Youden’s index; PPV, positive predictive value; NPV, negative predictive value. ^*a*^Cut off score with the highest Youden’s index value. ^*b*^PPV and NPV was calculated based on the prevalence from the research data.*

### Item Response Theory Analyses

**FIGURE 3:**
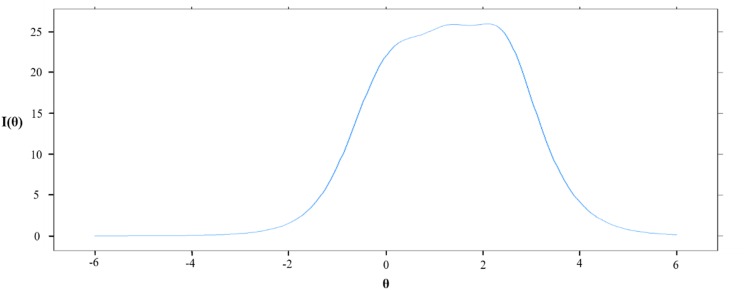
Test information curveof BDI-II. I(θ): Information value, θ: level of depression expressed in terms of standard deviation.

Item responses theory was applied to evaluate the test information function of BDI-II. The TIC is presented in Figure 3. The TIC represents how much information BDI-II provides at a certain level of depression. As presented in Figure 3, the BDI-II offered the most information with the lowest standard error of measurement at a depression level around 0–2.5 SD above the mean (Table 6) and forms a flat, plateau-like line, which indicates BDI-II is more suitable for testing severity evaluation (see Supplementary Table S3).

**TABLE 6:** Information Value for Each Area.

Θ area	Information	Proportion (%)
−2∼-1.5	1.24	1.17
−1.5∼-1	2.92	2.76
−1∼-0.5	6.01	5.69
−0.5∼0	9.60	9.08
0∼0.5	11.71	11.07
0.5∼1	12.35	11.68
1∼1.5	12.87	12.17
1.5∼2	12.91	12.21
2∼2.5	12.80	12.10
2.5∼3	10.51	9.94
Total area	105.74	100

## Discussion

This study aimed to examine the psychometric properties (e.g., reliability, factor structures, other construct validity) and diagnostic screening utility with optimal cutoff scores of the BDI-II within the framework of QUADAS-2. In addition, we investigated whether BDI-II would be appropriate as a severity or screening tool, using the IRT among Korean adult samples.

The Korean version of BDI-II showed an excellent level of internal consistency and item homogeneity and convergent validity. Cronbach’s alpha coefficient of 0.946 from this research was consistent or higher than those reported in the previous review of the internal consistency of the BDI-II among medical patients (i.e., ranging from 0.84 to 0.94) (Wang and Gorenstein, 2013). The Korean version of BDI-II also showed high correlations with other depression measures (i.e., PHQ-9 and CES-D), and GAD-7, which is also consistent with the previous comprehensive review of BDI-II (Wang and Gorenstein, 2013) and BDI-II validation study among adolescents in Korea (Lee et al., 2017). While analyzing individual items, all the original 21 BDI-II items remained. The item-total correlations of each item score with the total score ranged from.546 to.761. Items that showed high correlation included worthlessness, loss of pleasure (*r* = 0.759), loss of interest (*r* = 0.758), and self-dislike (*r* = 0.754), and items that showed lowest correlation included loss of libido (*r* = 0.546), changes in sleeping (*r* = 0.611), and changes in appetite (*r* = 0.601). Although it was reported that East Asians tend to express their depressive symptoms with somatic complaints (Yoo and Skovholt, 2001), somatic symptoms had the lowest correlations with BDI-II total scores. It is speculated that the lowest correlations might reflect the phenomenon that Koreans tend to report somatic symptoms not only for their depressive symptoms but also various psychiatric or health conditions. In addition, in our study, non-clinical samples had higher scores on the somatic symptoms than other depressive symptoms, indicating that somatic symptoms would be less depression specific.

In previous studies conducted with Western samples, the factor structure of the BDI-II was reported as a 2-factor model with somatic-affective factor and cognitive factor (Beck et al., 1996). However, in studies conducted in East Asian countries, a 3-factor model was suggested in adolescent samples (Wu and Huang, 2014; Lee et al., 2017). The somatic-affective factor of the 2-factor model was divided into “performance difficulty (PD)” and “somatic (SM)” factor. The 3-factor model was replicated in our Korean adult samples. In adolescent samples, the PD factor was first proposed to reflect the perception that adolescents are under the control of authority such as parents pursuing autonomy and competence, and experience conflicting demands arising from family, school, and peer groups (Byrne and Baron, 1993; Byrne et al., 1995). In the East Asian samples, the 3-factor model might have a better fit because individuals in East Asia seem to express depression with symptoms such as agitation, irritability, and concentration difficulty that occur when experiencing excessive pressure for achievement (Lee et al., 2017).

To assess the usefulness of BDI-II as a screening tool, optimal cut-off points for Korean adult population were suggested. Compared to the original criteria suggested by Beck et al. (1996), the 23-point cut-off score showed better performance detecting MDD than the moderate (score of 20) or severe (score of 29) criteria. For detecting DD, the 17-point cut-off score showed the best result. This result also showed better performance than the original mild level criterion (score of 14) (Beck et al., 1996). Based on these results, it seems reasonable to use 23 points as a criterion for moderate depression and 17 as a criterion for mild depression when measuring depression in the Korean population. The MDD group was strictly limited to people who were in current major depressive episodes, whereas the DD group included persistent depressive disorder (PDD) and depressive disorder not otherwise specified, as well as cases who were fully remitted from depressive disorder but still on medication. This interpretation is supported by previous studies. One study that measured the severity of college students at a student counseling center suggested 16 points for a mild cut-off score and 24 points for a moderate cut-off score (Sprinkle et al., 2002). Other studies reporting BDI-II cut-offs for the Korean population also support our data. Research by Sung et al. (2008) utilized the HDRS mild level as an index test reported 18 as a cut-off score, which is close to our DD cut-off score. Another study (Lim et al., 2011) that recruited MDD patients also suggested 22 as a cut-off score.

Finally, IRT analysis was used to determine whether BDI-II was more suitable as a screening tool or severity rating tool. IRT analysis suggested that BDI-II could offer equivalent information value from an average depressed population (where θ is 0) to a severely depressed population (where θ is 3), which is excellent for a severity rating tool, as mentioned earlier. This result is in line with a previous study (Brouwer et al., 2013) that conducted an IRT analysis of the BDI-II. A study by Brouwer et al. (2013) reported flat-looking TIC graphs for the range θ = 0–2, and argued that this may be more advantageous for detecting changes in depression in the clinical field. These results of the IRT analysis of BDI-II are consistent with the original intent of developing the BDI-II scale, which was to measure the depth of depression rather than simply presenting a single cut-off point (Beck et al., 1996). This suggests that BDI may be more useful for measuring depressive severity in clinical populations and for measuring depressive severity as an index of treatment responses.

Some limitations should be noted. In the present study, instead of using the Structured Clinical Interview for DSM-5 (SCID-5) which is regarded as golden standard for the diagnosis, M.I.N.I. was used as a reference test to confirm the compatibility of the BDI-II as a screening tool. Even though a trained psychologist administered the structured diagnostic interview, the M.I.N.I. was designed to reduce false negatives to avoid missing cases with actual illnesses (Sheehan et al., 1998). Therefore, it is possible to over-diagnose with the M.I.N.I., which might have affected the sensitivity or specificity of the BDI-II. Thus, in a future study, the results of the current study must be replicated by using different reference tests such as the SCID-5.

Since this study recruited samples from two different settings (hospitals and online advertisement) with different methods (consecutive sampling, random sampling), although factor structures of the BDI-II in each setting were identically favorable for a 3-factor model, future studies should identify whether participants recruited from online advertisements have distinctive characteristics from off-line hospital visitors. Lastly, this study provided test information value and item characteristics on the results of IRT analysis (see Supplementary Figures S1, S2). A future study might identify the best performing items of the BDI-II given the Korean population’s response style to and characteristic of each item of the BDI-II.

Despite the aforementioned limitations, the current study was the first validation study with adult Korean samples using the Korean version of the BDI-II with a formal license. This study was conducted rigorously in accordance with the QUADAS-2 framework, a system for evaluating screening tools. In addition, a relatively large sample of more than 1,000 people was used, and a cut-off score most appropriate for Korean people was calculated through a diagnostic interview with every single study participant. Finally, in addition to providing one single cut-off score, the IRT analysis suggested that the BDI-II may be a more appropriate tool for rating severity rather than screening.

## Data Availability Statement

The datasets generated for this study are available on request to the corresponding author.

## Ethics Statement

The studies involving human participants were reviewed and approved by the Korea University Institutional Review Board. The patients/participants provided their written informed consent to participate in this study.

## Author Contributions

KP, EJ, S-HL, and K-HC contributed to the conception and design of the study. K-HC supervised the overall study process. KP and SY performed the data analysis. KP wrote the first draft of the manuscript. KP, EJ, SY, and S-HL contributed to the acquisition of data. All authors contributed to the manuscript revision, read, and approved the submitted version.

## Conflict of Interest

The authors declare that the research was conducted in the absence of any commercial or financial relationships that could be construed as a potential conflict of interest.

## Footnotes

1 http://www.phqscreeners.com
