# Supplementary material for: Diagnostic Utility and Psychometric Properties of the Beck Depression Inventory-II Among Korean Adults
Source: Front Psychol. 2020 Jan 21;10:2934. doi: 10.3389/fpsyg.2019.02934 (PMC6985267; doi:10.3389/fpsyg.2019.02934)
Supplement: Supplementary file 1 [file Data_Sheet_1.PDF]

Suppl Table 1. Summary of CFA fit indices for online advertisement sample

Model tested	Fit indices								
	χ^2	AIC	BIC	aBIC	CFI	TLI	SRMR	RMSEA	90% CI
3-Factor Model	533.972***(*df* = 186)	20941.00	21225.70	21016.18	0.931	0.922	0.041	0.058	0.052–0.064
2-Factor Model	657.990*** (*df* = 188)	21061.02	21337.09	21133.92	0.907	0.896	0.051	0.067	0.062–0.073

Note. AIC: Akaike information criterion, BIC: Bayesian information criterion, aBIC: Sample-size adjusted BIC, CFI: Comparative fit index, TLI: Tucker-Lewis Index, SRMR: Standardized root mean squared residual, RMSEA: Root mean square error of approximation, CI: Confidence interval, ***< 0.001,

Suppl Table 2. Summary of CFA fit indices for hospital visitor sample

Model tested	Fit indices								
	χ^2	AIC	BIC	aBIC	CFI	TLI	SRMR	RMSEA	90% CI
3-Factor Model	598.591***(*df* = 186)	22678.87	22967.96	22758.43	0.951	0.944	0.035	0.061	0.056–0.067
2-Factor Model	636.509***(*df* = 188)	22712.79	22993.11	22789.93	0.946	0.940	0.037	0.064	0.058–0.069

Note. AIC: Akaike information criterion, BIC: Bayesian information criterion, aBIC: Sample-size adjusted BIC, CFI: Comparative fit index, TLI: Tucker-Lewis Index, SRMR: Standardized root mean squared residual, RMSEA: Root mean square error of approximation, CI: Confidence interval, ***< 0.001,

Suppl Table 3. Information value for each area for each group

Θ area	Online advertisement sample		Hospital visitor sample	
	Information	Proportion (%)	Information	Proportion (%)
−2 ~ −1.5	1.32	1.44	1.07	0.89
−1.5 ~ −1	2.67	2.92	2.99	2.49

−1 ~ −0.5	4.87	5.33	6.98	5.81
−0.5 ~ 0	7.29	7.97	11.82	9.83
0 ~ 0.5	8.70	9.51	15.01	12.48
0.5 ~ 1	9.02	9.81	16.26	13.5
1 ~ 1.5	9.31	10.19	16.90	14.06
1.5 ~ 2	9.24	10.11	17.05	14.18
2 ~ 2.5	9.25	10.11	15.18	12.62
2.5 ~ 3	9.04	9.89	9.51	7.91
Total area	91.422	100	120.20	100

Suppl Figure 1. Item characteristic curve for each item of BDI-II.

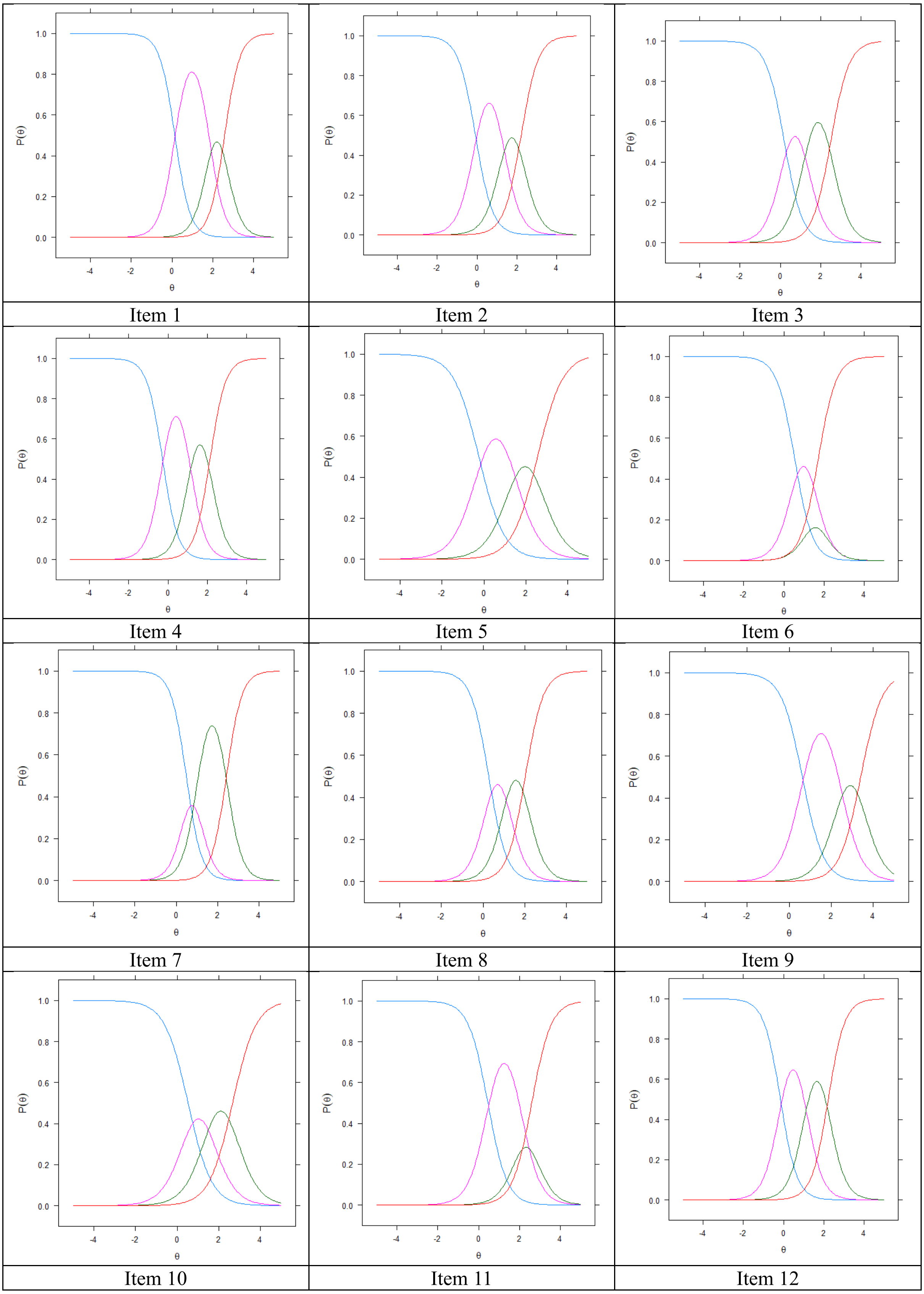

Figure continued

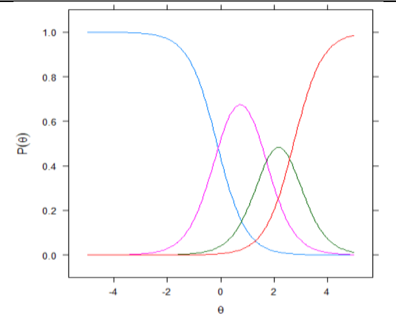

Item 13

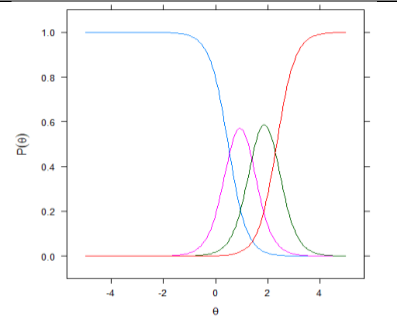

Item 14

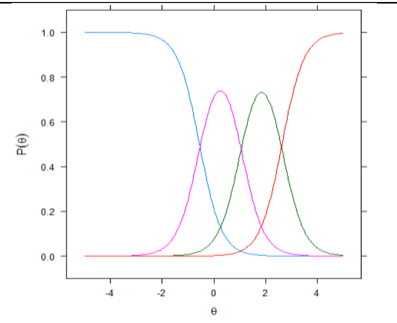

Item 15

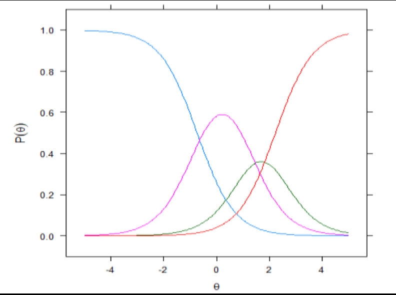

Item 16

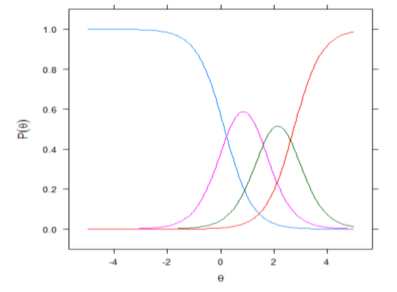

Item 17

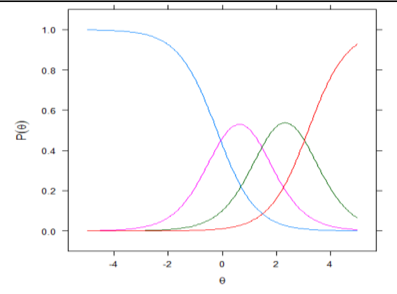

Item 18

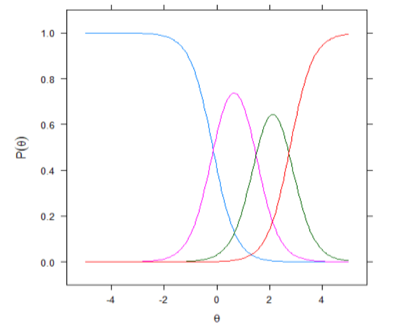

Item 19

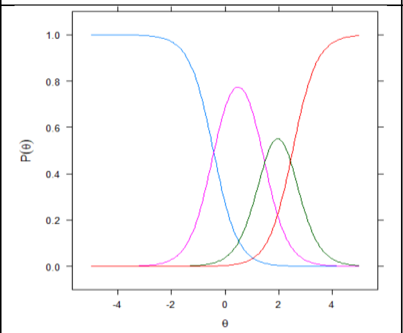

Item 20

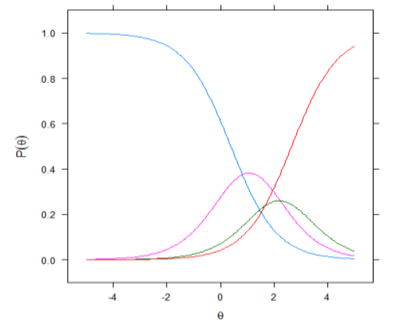

Item 21

Suppl Figure 2. Item information curve for each item of BDI-II.

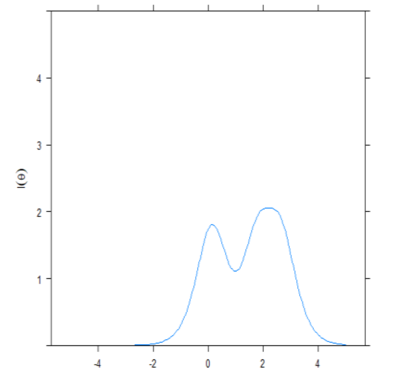	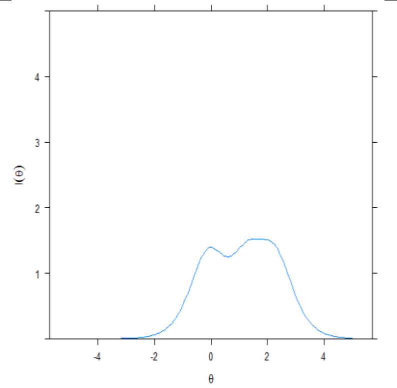	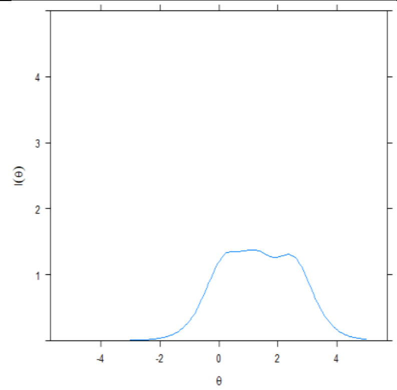
Item 1	Item 2	Item 3
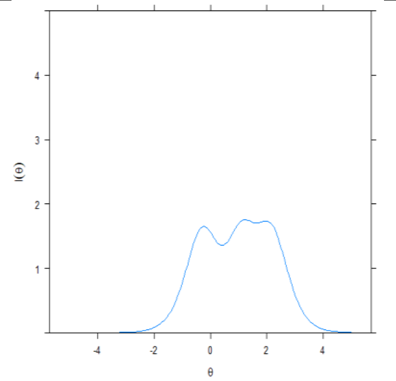	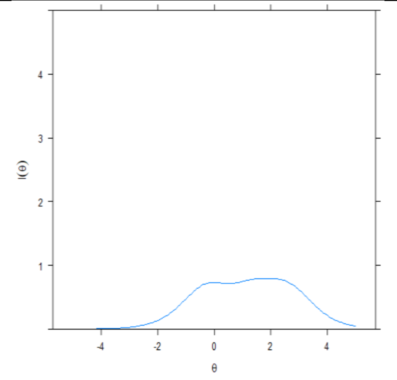	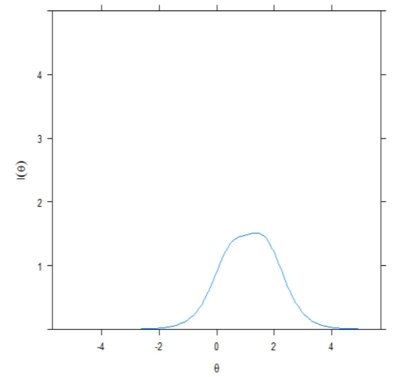
Item 4	Item 5	Item 6
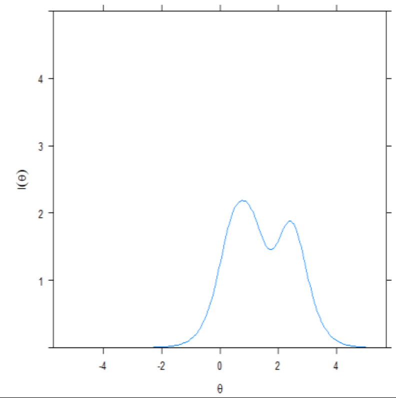	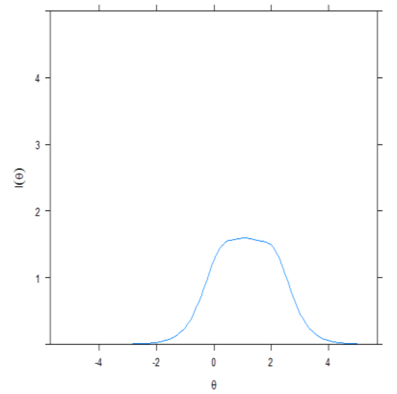	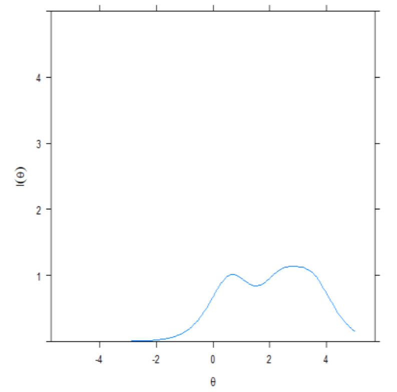
Item 7	Item 8	Item 9
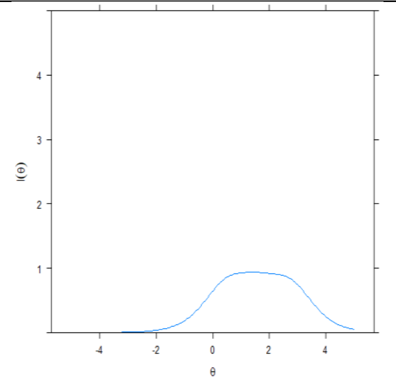	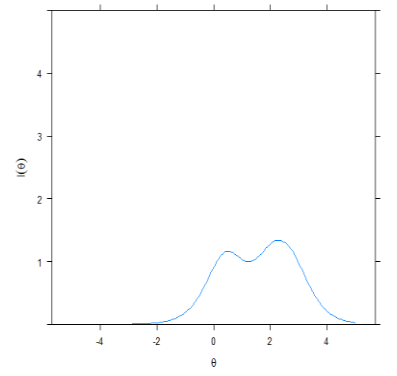	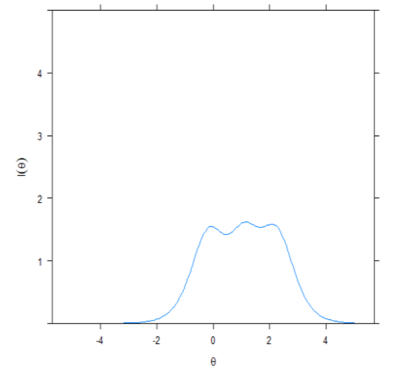
Item 10	Item 11	Item 12

Figure continued

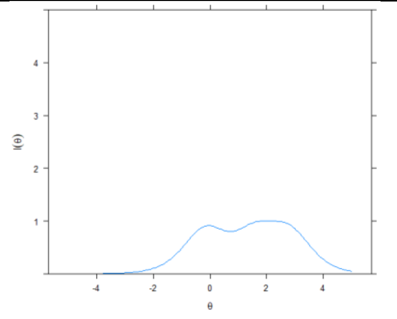

Item 13

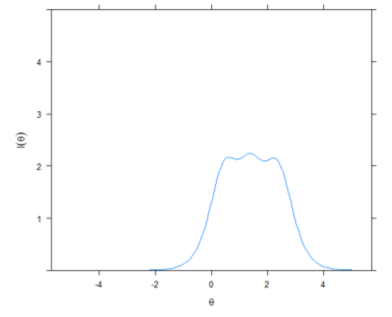

Item 14

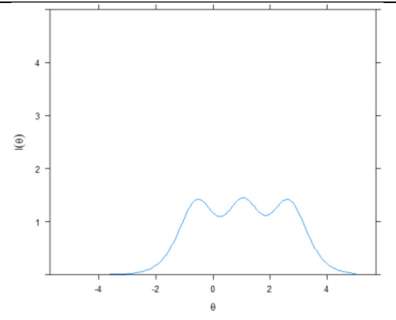

Item 15

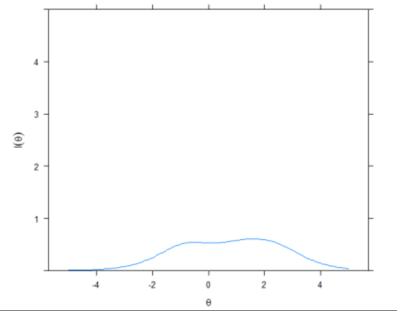

Item 16

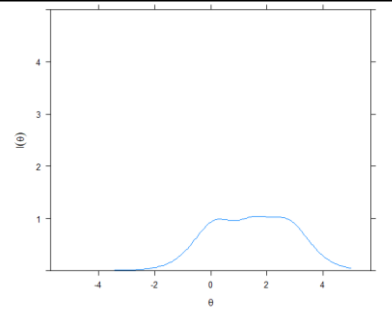

Item 17

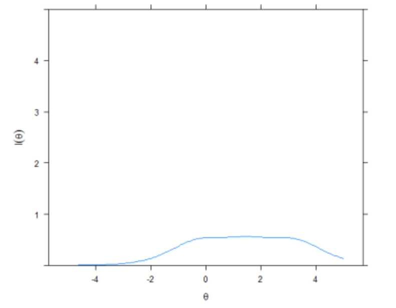

Item 18

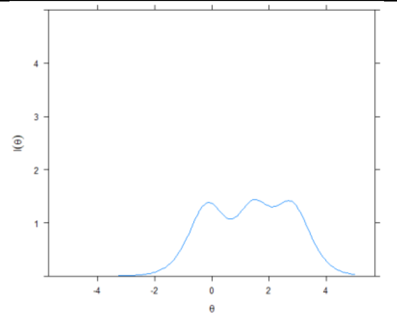

Item 19

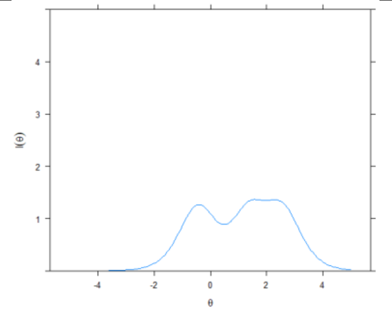

Item 20

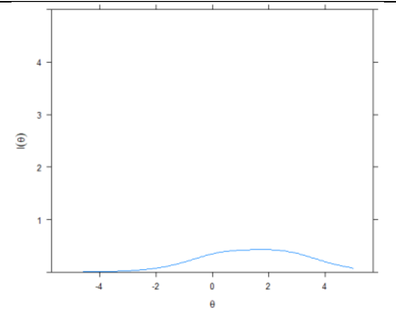

Item 21
